# SEOM clinical guidelines in metastatic breast cancer 2015

**DOI:** 10.1007/s12094-015-1476-7

**Published:** 2015-12-18

**Authors:** J. Gavilá, S. Lopez-Tarruella, C. Saura, M. Muñoz, M. Oliveira, L. De la Cruz-Merino, S. Morales, I. Alvarez, J. A. Virizuela, M. Martin

**Affiliations:** Servicio de Oncología Médica, Fundación Instituto Valenciano de Oncología, Valencia, Spain; Servicio de Oncología Médica, Hospital General Universitario Gregorio Marañón, Instituto de Investigación Sanitaria Gregorio Marañón (IiSGM), Madrid, Spain; Servicio de Oncología Médica, Hospital Vall d’ Hebron, Barcelona, Spain; Servicio de Oncología Médica, Hospital Clinic I Provincial, Barcelona, Spain; Servicio de Oncología Médica, Complejo Hospitalario Regional Virgen Macarena, Seville, Spain; Servicio de Oncología Médica, Hospital Universitari Arnau de Villanova de Lleida, Lleida, Spain; Servicio de Oncología Médica, Hospital Donostia-Donostia Ospitalea, Donostia, Spain; Instituto de Investigación Sanitaria Gregorio Marañón, Universidad Complutense, Madrid, Spain

**Keywords:** Metastatic breast cancer, Clinical guidelines, Chemotherapy, Hormonal therapy, Anti-HER2 therapy

## Abstract

Metastatic breast cancer is essentially an incurable disease. However, recent advances have resulted in a significant improvement of overall survival. The SEOM guidelines are intended to make evidence-based recommendations on how to manage patients with metastatic breast cancer to achieve the best patient outcomes based on a rational use of the currently available therapies. To assign a level of certainty and a grade of recommendation the United States Preventive Services Task Force guidelines methodology was selected as reference.

## Introduction

Metastatic breast cancer is essentially an incurable disease. However, recent advances in the medical treatment of patients with some of the most frequent subtypes (particularly the luminal and HER2 subtypes) have resulted in a significant improvement of the median overall survival (OS) of these patients; median OS figures of around 5 years have been reported in the most recent randomized trials in patients with these subtypes.

This guide is intended to make evidence-based recommendations on how to manage patients with metastatic breast cancer to achieve the best patient outcomes by means of a rational use of the currently available therapies.

## Methodology

The SEOM Guidelines have been developed with the consensus of ten breast cancer oncologists from the cooperative groups GEICAM (Spanish Breast Cancer Research Group) and SOLTI (Spanish Collaborative Group for the Study, Treatment and Other Experimental Strategies in Solid Tumors). To assign a level of certainty and a grade of recommendation to the different statements described in the clinical guidelines, the United States Preventive Services Task Force (USPSTF) guidelines methodology was selected as reference [1] (Table 1).

**Table 1:** Strength of recommendation and level of certainty

Category (grade)	Definition
Strength of recommendation	
A	The USPSTF recommends the service. There is high certainty that the net benefit is substantial
B	The USPSTF recommends the service. There is high certainty that the net benefit is moderate or there is moderate certainty that the net benefit is moderate to substantial
C	The USPSTF recommends against routinely providing the service. There may be considerations that support providing the service in an individual patient. There is moderate or high certainty that the net benefit is small
D	The USPSTF recommends against the service. There is moderate or high certainty that the service has no net benefit or that the harms outweigh the benefits
I statement	The USPSTF concludes that the current evidence is insufficient to assess the balance of benefits and harms of the service. Evidence is lacking, of poor quality or conflicting, and the balance of benefits and harms cannot be determined
Level of certainty	
High	The available evidence usually includes consistent results from well-designed, well-conducted studies in representative primary care populations. These studies assess the effects of the preventive service on health outcomes. This conclusion is, therefore, unlikely to be strongly affected by the results of future studies
Moderate	The available evidence is sufficient to determine the effects of the preventive service on health outcomes, but confidence in the estimate is constrained by factors such as the number, size, or quality of individual studies; inconsistency of findings across individual studies; limited generalizability of findings to routine primary care practice; or lack of coherence in the chain of evidence. As more information becomes available, the magnitude or direction of the observed effect could change, and this change may be large enough to alter the conclusion
Low	The available evidence is insufficient to assess effects on health outcomes. Evidence is insufficient because of: the limited number or size of studies; important flaws in study design or methods; inconsistency of findings across individual study gaps in the chain of evidence; findings not generalizable to routine primary care practice; or a lack of information on important health outcomes. More information may allow an estimation of effects on health outcomes

## Goal of the treatment

Metastatic breast cancer (MBC) is a treatable but virtually incurable disease [2]. The two main goals of care in MBC are to improve survival and to optimize quality of life [3].

- All MBC patients should be offered comprehensive, individualized, up-to-date, and easy to understand information about their disease and its management (level of certainty: high; strength of recommendation: A).
- As soon as MBC is diagnosed, patients should also be offered appropriate multidisciplinary care, including symptom-related intervention (level of certainty: high; strength of recommendation: A).
- Survival may be greater for patients treated in specialized institutions. Management of MBC patients by specialized multidisciplinary teams in specialized institutions should be encouraged (level of certainty: moderate; strength of recommendation: B).
- Investigation remains a priority in this setting. Participation in well-designed, independent, prospective trials should be offered to all eligible patients, whenever possible (level of certainty: high; strength of recommendation: A).

## Determination of the metastatic spread and retesting of biomarkers in recurrent disease

There is a general consensus that an adequate study of tumor extension with histological confirmation of metastatic lesions whenever possible is recommendable in the initial management of MBC.

- Physical examination, blood parameters, body CT, MRI and bone scintigraphy are the recommended methods of study of extension (level of certainty: moderate; strength of recommendation: B).
- The role of PET/CT in the determination of metastatic spread is controversial [4, 5] (level of certainty: low; strength of recommendation: C).
- The routine use of tumoral markers in the follow-up of breast cancer is controversial (level of certainty: low; strength of recommendation: C).
- Circulating tumor cells (CTC) enumeration in MBC is not recommended as a routine in disease assessment and monitoring. CTC have prognostic but not predictive value [6] (level of certainty: low; strength of recommendation: I statement).
- Histopathological assessment of the metastasis is recommendable since a change of the phenotype of metastases with respect to the primary tumor has been described. These changes can lead to a modification of the therapeutic approach [7–9] (level of certainty: high; strength of recommendation: A).

## Evaluation of response to therapy in advanced breast cancer

The evaluation of response to therapy should be done after an interval treatment of 2–3 months to rule out progression of the disease. In patients with non-aggressive disease, i.e., soft tissue or bone metastasis, the time to evaluation could be longer than in patients with visceral and aggressive disease.

- The utility of serum tumor markers (TM) (CEA, CA15.3 and CA27-29) assessment in monitoring response to treatment in ABC patients is controversial. Treatment decisions should not be based just in variations of TM serum levels. In patients with non-measurable disease, an increase in TM levels may be indicative of treatment failure [10, 11] (level of certainty: moderate; strength of recommendation: A).
- Radiologic assessment: The RECIST criteria (intended for response evaluation in clinical trials, but also useful in routine clinical practice) were updated in 2009 [12]. CT or MRI is the best method to measure metastasis lesions (level of certainty: low; strength of recommendation: C).
- Although combination of FDG-PET and CT offers more information than the conventional imaging, the utility of PET/CT in the monitoring of breast cancer metastasis is controversial [13] (level of certainty: low; strength of recommendation: C).

## Treatment of HER2-positive MBC

### First-line therapy

- Specific anti-HER2 treatment should be offered as soon as possible to all patients with metastatic HER2-positive breast cancer (level of certainty: high; strength of recommendation: A).
- First-line treatment with trastuzumab in combination with chemotherapy (especially taxanes), is associated to improvement of: response rate, progression-free survival (PFS), time to progression and OS, versus only chemotherapy [14] (level of certainty: high; strength of recommendation: A).
- In postmenopausal patients with hormone receptor-positive (HR) and HER2-positive tumors, the combination of aromatase inhibitors and an anti-HER2 agent (trastuzumab or lapatinib) has shown an increase in the response rate and the progression-free survival rate, but not in overall survival versus hormone therapy alone [15–17]. However, the response rate and PFS with these combinations seem inferior to the ones reached with chemotherapy plus trastuzumab and, therefore, should be limited to low-risk patients (i.e., patients without visceral disease) (level of certainty: moderate; strength of recommendation: B).
- Based on the benefits shown in terms of the response rate, progression-free survival and overall survival in a well-conducted Phase III study (the CLEOPATRA trial), the current treatment of choice, provided there are no contraindications, should be a combination of docetaxel, trastuzumab and pertuzumab [18] (level of certainty: high; strength of recommendation: A). Replacing docetaxel with paclitaxel or vinorelbine may be considered in certain circumstances [19] (level of certainty: low; strength of recommendation: C).
- In patients with relapse after adjuvant trastuzumab, there is limited scientific evidence on the best treatment option, since few patients with these characteristics were included in the CLEOPATRA trial. In relapses occurring more than 1 year after completion of adjuvant trastuzumab, the combination of docetaxel, trastuzumab, and pertuzumab may be considered; whereas with relapses of 6–12 months, there is stronger evidence in favor of T-DM1 (level of certainty: moderate; strength of recommendation: B).

### Second-line therapy

- Several studies have shown that there is a benefit in continuing with second-line anti-HER2 therapy, after progression during or following first-line treatment with trastuzumab [20–24] (level of certainty: high; strength of recommendation: A).
- T-DM1 was superior to lapatinib plus capecitabine (prior second-line standard) in terms of response rate, PFS and OS in patients pretreated with either first-line trastuzumab combinations or early relapses after trastuzumab adjuvant therapy [23]. T-DM1 is the preferred second-line option for this population (level of certainty: high; strength of recommendation: A).
- Lapatinib plus capecitabine can be a good second-line option for patients with contraindications for T-DM1 (level of certainty: moderate; strength of recommendation: B).

### Third-line and further therapy

- Patients with advanced HER2-positive breast cancer, who have been treated with two or more lines of anti-HER2 therapy, may benefit from a third or further line of anti-HER2 [22–24] (level of certainty: high; strength of recommendation: A).
- T-DM1 may be considered a new standard for the treatment of patients with advanced HER2-positive breast cancer previously treated with anti-HER2 therapy (including trastuzumab, lapatinib and pertuzumab), with or without chemotherapy (level of certainty: high; strength of recommendation: A).
- The combination of lapatinib plus trastuzumab in patients progressing on trastuzumab showed a higher PFS and OS versus lapatinib alone. The benefit was more notable in the sub-group of HR-negative patients [22] (level of certainty: moderate; strength of recommendation: B).
- The optimal number of lines of anti-HER2 therapy for metastatic breast cancer is currently unknown, although available data suggest benefits are maintained in third-line and further therapy (level of certainty: moderate; strength of recommendation: B).

**Fig. 1:**
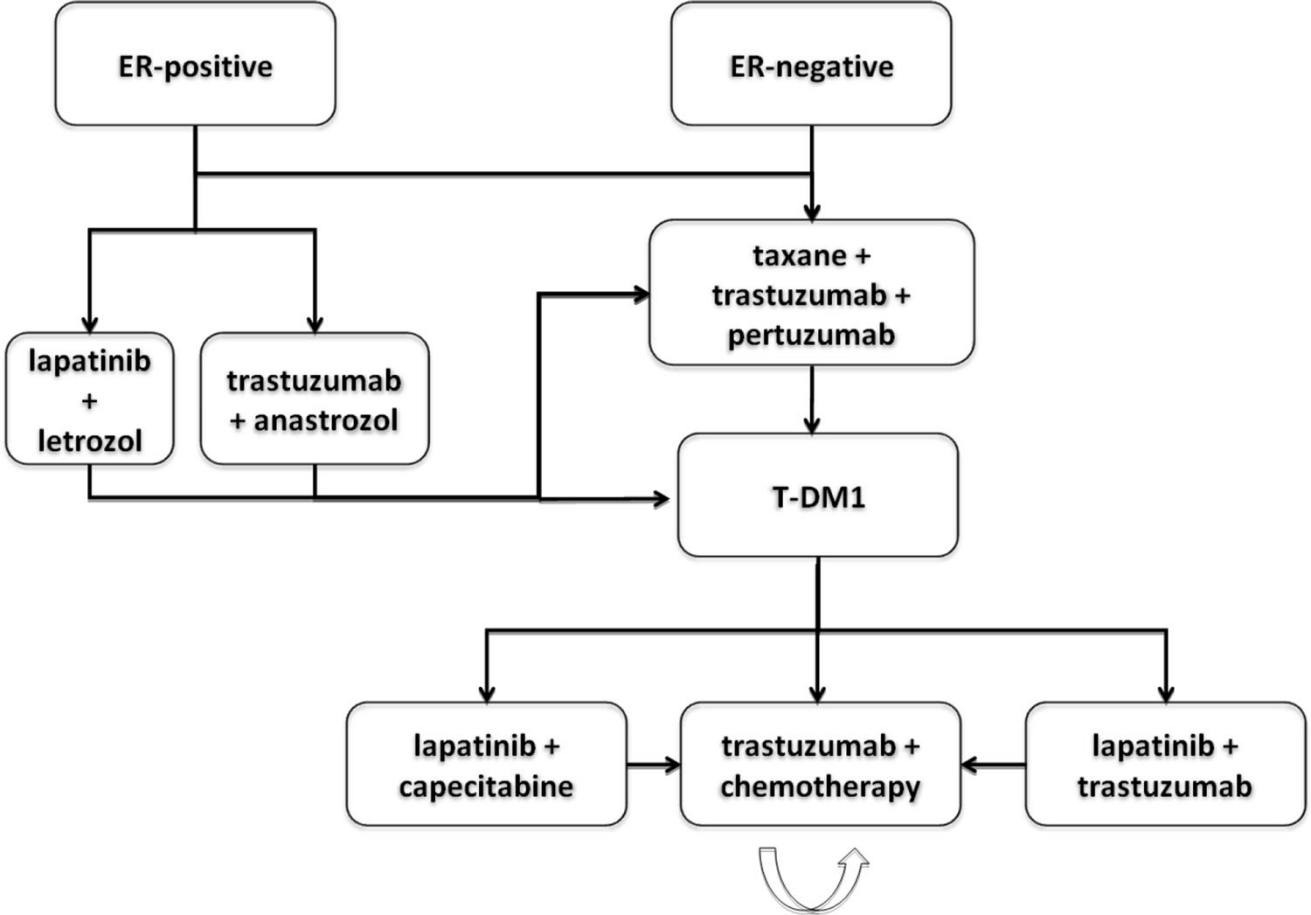
Therapeutic algorithm for HER2-positive metastatic breast cancer

Figure 1 shows an algorithm for the treatment of HER2-positive metastatic breast cancer, in accordance with the prior recommendations.

## Treatment of hormone-sensitive HER2-negative MBC

- Endocrine therapy (ET) is the recommended first option for this group of patients; only those patients presenting with rapidly progressive visceral metastasis are candidates to chemotherapy as first option (level of certainty: high; strength of recommendation: A). Available endocrine agents and their mechanisms of action are listed in Table 2 [25].

**Table 2:** Common classes of endocrine therapy

Mechanism of action	Class	Agent
Estrogen receptor blockage	SERM	Tamoxifen, toremifene
Estrogen receptor downregulation	SERD	Fulvestrant
Estrogen deprivation	Ovarian ablation	Surgery, radiation
	Ovarian suppression with GnRH	Goserelin, triptorelin, leuprolide
Aromatase inhibition	NSAI	Anastrozole, letrozole
	SAI	Exemestane
Unknown	Progestins	Megestrol acetate, medroxyprogesterone acetate
	High-dose estrogens	Diethylstilbestrol (DES)

Mechanism of action

*SERM* selective estrogen receptor modulator, *SERD* selective estrogen receptor downregulator (fulvestrant 500 mg/month with loading doses is the recommended schedule), *GnRH* gonadotropin hormone-releasing hormone, *NSAI* non-steroidal aromatase inhibitors (third generation), *SAI* steroidal aromatase inhibitors (third generation)
- Clinical benefit (CB) to ET is defined as the achievement of an objective response or disease stabilization lasting more than 24 weeks. The achievement of CB is predictor of survival among metastatic patients on ET and also predicts benefit to the subsequent ET.
- Nearly one-third of patients with ER/PR-positive tumors have primary resistance and nearly all of them eventually develop secondary resistance to ET. Endocrine resistance have been defined as primary [relapse while on the first 2 years of adjuvant ET, or progression disease (PD) within first 6 months of first-line ET for MBC, while on ET] or secondary or acquired (relapse while on adjuvant ET but after the first 2 years, or a relapse within 12 months of completing adjuvant ET, or PD ≥6 months after initiating ET for MBC, while on ET) [3].
- The selection of the optimal agent for ET in MBC is driven by menopausal status, prior ET therapy and patient co-morbidities.

### Premenopausal women

- First-line treatment: Ovarian ablation or suppression (OA) or tamoxifen has similar results, the combination of OA plus tamoxifen offers better results than OA alone and is considered the best option [25] (level of certainty: high; strength of recommendation: A).
- Second-line treatment: For patients progressing on tamoxifen or GnRH agonists, switching to the opposite is an acceptable option, as well as the combination of both (level of certainty: low; strength of recommendation: B).
- Although there is no phase III data exploring the value of the combination of OA and AI or fulvestrant, these combinations can be used in patients progressing on OA + tamoxifen (level of certainty: low; strength of recommendation: B).
- A randomized phase III trial has shown that the combination of ET (fulvestrant in postmenopausal women, fulvestrant plus OS in premenopausal women) and palbociclib improves PFS vs ET alone. OA plus fulvestrant plus palbociclib is, therefore, a second-line option for premenopausal women, although palbociclib is not yet available for prescription in Spain [26] (level of certainty: high; strength of recommendation: A).

### Postmenopausal women

#### First-line therapy (59)

- Anastrozole, letrozole and exemestane are superior to tamoxifen in PFS in patients without prior ET or only adjuvant tamoxifen (level of certainty: high; strength of recommendation: A).
- Fulvestrant (250 mg/monthly) is equivalent to tamoxifen. But this dose is now considered suboptimal. Recommended dose is fulvestrant 500 mg/monthly (level of certainty: high; strength of recommendation: A).
- The combination of anastrozole and fulvestrant (250 mg/monthly) versus anastrozole alone was explored in two randomized phase III trials, with contradictory results. This combination cannot be currently recommended for routine use (level of certainty: low; strength of recommendation: B).
- Fulvestrant (LD 500 mg) was superior to anastrozole in PFS and OS according to a randomized phase II trial. Data from the phase III trial are pending (level of certainty: low; strength of recommendation: B).
- Letrozol + Palbociclib is superior to letrozole according to a randomized phase II trial (PFS). Data from the phase III trial are pending (level of certainty: moderate; strength of recommendation: B).
- Fulvestrant 500 mg was superior to fulvestrant 250 mg in PFS and OS in patients with prior adjuvant ET and/or ET for metastatic disease and could be a first-line ET option in patients with adjuvant AI (level of certainty: moderate; strength of recommendation: B).
- Considering these data, AI is the first-line option with the best evidence in patients without prior adjuvant AI (level of certainty: high; strength of recommendation: A). In patients treated with adjuvant AI, fulvestrant 500 mg is also a good option (level of certainty: moderate; strength of recommendation: B).

#### Second-line therapy

- In patients progressing on tamoxifen, AIs were equivalent or modestly superior to progestins but much better tolerated and fulvestrant 250 mg was equivalent to anastrozole.
- In patients progressing on a non-steroidal AI exemestane and fulvestrant (250 mg) were equivalent.
- Fulvestrant 500 mg was superior to fulvestrant 250 mg in PFS and OS in a phase III trial including around 50 % of patients with only adjuvant ET.
- Exemestane plus everolimus was superior to exemestane in PFS (but not OS) in a phase III trial including mainly patients with prior therapy for metastases (around 80 % of total).
- Palbociclib plus fulvestrant was superior to fulvestrant in PFS in a phase III trial. Palbociclib is not yet available for prescription in Spain.
- Fulvestrant 500 mg or exemestane + everolimus is the best currently available option after progression to a non-steroidal AI (level of certainty; high strength of recommendation: A). These two options could be used in sequence, but the optimal order is unknown.

### Late endocrine lines

- There is very limited information from prospective trials in patients with prior exposition to AI, tamoxifen fulvestrant and exemestane–everolimus. In cases where a positive effect has been achieved with prior ET, progestins (megestrol acetate or medroxyprogesterone) androgens and high-dose estrogens could be used in selected highly sensitive cases.

#### Chemo-endocrine therapy

- There is no clear evidence that concomitant use of ET plus chemotherapy results in improvement on OS. Therefore, this combination should be discouraged outside a clinical trial (level of certainty: low; strength of recommendation: D).

### ET as maintenance after chemotherapy

- Phase II trials and observational studies have suggested that patients with HR-positive tumors treated with chemotherapy in first-line could have an improvement in PFS with maintenance ET. Therefore, ET after chemotherapy can be considered as a reasonable option in these patients [3] (level of certainty: low; strength of recommendation: C).

### Chemotherapy (CT) for HR-positive MBC

CT is the standard treatment for HR-positive MBC once hormonal therapy is not working any longer. Anthracyclines, taxanes, vinorelbine, capecitabine, gemcitabine and eribulin are available options. The choice of the strategy and cytotoxic agents must be considered individually. In general, sequencing single-agent chemotherapy is preferred [27], limiting combination therapies for patients with aggressive, symptomatic or life-threatening disease [3] (level of certainty: high; strength of recommendation: A).

#### First-line treatment

- Anthracyclines or taxanes, either alone or in form of combinations, are considered the first-line chemotherapy of choice, particularly in patients without prior adjuvant chemotherapy or with late relapses (level of certainty: high; strength of recommendation: A).
- The combination of bevacizumab plus taxanes improves PFS and ORR, but not OS versus chemotherapy alone and should also be considered a first-line chemotherapy option [28–30] (level of certainty: moderate; strength of recommendation: C).
- In patients pretreated with adjuvant taxanes and anthracyclines, other options such as vinorelbine [31, 32] and capecitabine [33] are also appropriate first-line chemotherapy options (level of certainty: moderate; strength of recommendation: B).

#### Second and further lines of chemotherapy

- A large number of agents have shown activity as second-line chemotherapy and beyond in HR-positive MBC and may be suitable for sequential treatment in selected patients. Among them, capecitabine, vinorelbine, gemcitabine, nab-paclitaxel, liposomal doxorubicin and eribulin are approved options and can be appropriate therapies [31, 34–37].
- In a phase III trial in patients pretreated with taxanes and anthracyclines, eribulin was not superior to the current standard capecitabine in PFS or OS.
- In another phase III trial, eribulin has shown a modest improvement in OS in patients with prior taxanes, anthracyclines and capecitabine. Therefore, it is the CT drug of choice in this population (level of certainty: high; strength of recommendation: A).
- Considering these data, capecitabine is the most recommendable first option for HR-positive MBC patients pretreated with taxanes and anthracyclines, while eribulin can be administered after progression on capecitabine (level of certainty: high; strength of recommendation: A).

## Treatment of triple-negative MBC

Triple-negative breast cancer (TNBC) is a somewhat heterogeneous entity, although at least 70 % of patients are of the basal-like subtype by PAM50 subtyping. Basal-like breast cancer is characterized by high proliferation (as measured by Ki67) and frequent p53 mutations. CT is the standard treatment for patients with TNBC [38].

### First-line treatment

- In patients that are CT naïve, anthracyclines or taxanes, either alone or in combinations, are considered as first-line treatment (level of certainty: high; strength of recommendation: A). This recommendation is also valid for patients with late recurrences (>1 year) after adjuvant anthracyclines and/or taxanes.
- In patients pretreated with adjuvant taxanes and anthracyclines, other options such as vinorelbine [31, 32] and capecitabine [33] are also available (level of certainty: moderate; strength of recommendation: B).
- The combination of bevacizumab plus taxanes improves PFS and ORR, but not OS versus chemotherapy alone in the subset of patients with TNBC and should also be considered a first-line option [28–30] (level of certainty: moderate; strength of recommendation: C).
- The role of platinum compounds in TNBC is under debate. These compounds significantly increase the pCR rate in TNBC patients (regardless of BRCA status) when added to conventional neoadjuvant CT [39] but have not been adequately tested in metastatic TNBC.
- Despite the absence of phase III data, the combination of carboplatin and gemcitabine has been accepted as an appropriate control arm by the EMA and the FDA in randomized trials and actually showed a significant activity (median PFS of around 5 months and median OS of around 1 year as first-line therapy) [40]. The combination is active in patients resistant to anthracyclines and taxanes and it is an acceptable option for these patients (level of certainty: low; strength of recommendation: B).
- In a phase III trial in patients with both TNBC- and BRCA-associated metastatic tumors, carboplatin and docetaxel were similarly effective in the overall population [41]. Carboplatin was superior to docetaxel in ORR and PFS in patients with BRCA-associated tumors and can be considered an option for these patients (level of certainty: moderate; strength of recommendation: B).
- Platinum salts, alone or in combinations could be an option as first-line chemotherapy for TNBC, although appropriate randomized trials are needed to further define the real interest of these drugs (level of certainty: moderate; strength of recommendation: B).

### Second and further lines of treatment

- There is no limit to the number of therapy lines to be proposed to metastatic TNBC patients, as long as a good quality of life is maintained.
- Capecitabine, vinorelbine, gemcitabine, nab-paclitaxel, liposomal doxorubicin and eribulin are approved drugs and can be appropriate options [31, 33–37].
- In a phase III trial, eribulin was not superior to capecitabine in terms of PFS and OS in the overall population of patients pretreated with anthracyclines and taxanes, but in the TNBC population the differences in OS were favorable to eribulin [35].

As there are few effective treatment options and TNBC is an aggressive disease, patients with this condition should be offered the participation in well-designed, independent, clinical trials testing new molecules and with a robust translational research plan.

## Treatment of central nervous system metastases

Diagnosis of brain metastases is associated with the shortest survival compared with other sites of metastatic disease in breast cancer [42]. A modified breast-graded prognostic assessment (GPA) index has been recently postulated (63). It integrates four simple clinical characteristics and may serve to guide further treatments in BMBC patients (Table 3). Good prognosis patients (index 2.5 or higher) could be candidates of aggressive local control with strategies like surgery and/or radiation therapy. On the other hand, poor prognosis patients (<2.0 in the modified breast-GPA index) might initially be managed in a more conservative way, with whole brain radiotherapy (WBRT) and symptomatic treatment. Anyway, the breast-GPA index for BMBC is just a complimentary tool in the decision-making process.

**Table 3:** Modified breast-GPA index for BMBC patients

Factor	0	0.5	1.0	1.5	2.0
Karnofsky score	≤50	60	70–80	90–100	–
Breast cancer subtype	Triple negative	Hormone receptor positive/HER2 negative	Hormone receptor negative/HER2 positive	Hormone receptor positive/HER2 positive	–
Age (years)	≥50	≤50	–	–	–
Number of brain metastases	≥3	1–3	–	–	–

### Local therapies

- Surgical management of BMBC can be an adequate option for high breast-GPA index, or in patients with 1–3 brain metastases and systemic disease under control [43]. Although impact on overall survival is not clear, whole brain radiation therapy (WBRT) after surgery of BMBC increases local tumor control and reduces intracranial relapses [44] (level of certainty: moderate; strength of recommendation: B).
- Stereotactic radiotherapy (SRS) is generally indicated in selected cases of oligometastatic disease (≤3 metastases) and can be considered an alternative to surgery [45] (level of certainty: moderate; strength of recommendation: B).
- Whole brain radiation therapy is associated with a moderate improvement on OS with respect to best supportive care [43]. WBRT is generally recommended for ≥3 metastases, and/or when lesions are higher than 3 cm or have a volume of ≥25 cm^3^ [46] (level of certainty: moderate; strength of recommendation: B).

### Systemic treatments

- The value of systemic treatments on local control of brain metastases is unclear (level of certainty: moderate; strength of recommendation: B).

#### Local therapy of the primary tumor in the novo MBC

- Two meta-analyses have found that surgery of the primary tumor in patients with metastatic disease at diagnosis was independently associated with a better survival [47, 48]. However, a selection bias cannot be ruled out.
- Two randomized trials have failed to show an improvement on OS by adding breast surgery to systemic therapy [49, 50].
- Therefore, routine breast surgery cannot be recommended in de novo metastatic breast cancer (level of certainty: moderate; strength of recommendation: I statement).

#### The role of surgery of extracranial metastasis in MBC

- Systemic therapy is the standard therapeutic approach for MBC patients but in selected cases surgery of distant metastasis could be considered after a balanced decision process.
- Oligometastatic disease in fit patients with long-term disease-free intervals or good response to previous systemic treatments could be considered as criteria for surgery with a curative intent (level of certainty: low; strength of recommendation: I statement).

#### The role of radiotherapy of extracranial MBC

- The role of radiotherapy (RT) in MBC can be palliative (i.e., uncontrolled pain, spinal cord compression or fractures) or with a radical intention in selected cases of oligometastatic disease.
- Radiotherapy (i.e., hypofractionated image-guided radiotherapy) can be an alternative to surgery as local therapy in some patients with oligometastatic disease who are not candidates for surgery (level of certainty: low; strength of recommendation: I statement).

## Treatment of bone metastases

- In prospective controlled trials, bisphosphonates (pamidronate, zoledronic acid) have shown to be effective in delaying complications of bone metastases from breast cancer and should be added to anti-tumor therapy in patients with breast cancer and bone metastases [51] (level of certainly high; strength of recommendation: A).
- In a large, randomized phase III study, denosumab was superior to zoledronic acid in delaying the time to first squeletal-related event (SREs: fractures, spinal cord compression, radiation to the bone or surgery) and the time to the first and subsequent SREs. Denosumab also significantly reduced the mean skeletal morbidity rate as compared with zoledronic acid. Therefore, denosumab is also an appropriate option, in conjunction with anti-tumor therapy, for the management of bone metastases from breast cancer [52] (level of certainly high; strength of recommendation: A).
- The most appropriate duration of anti-resorptive therapy (bisphosphonates, denosumab) in breast cancer patients with bone metastases is unknown. The experience with zoledronic acid and denosumab beyond two years of administration is very limited (level of certainty moderate; strength of recommendation: B).
